# Crystal structure of poly[μ-diphen­yl(pyridin-4-yl)phosphane-κ^2^*N*:*P*-μ-tri­fluoro­acetato-κ^2^*O*:*O*′-silver(I)] from synchrotron data

**DOI:** 10.1107/S2056989025011302

**Published:** 2026-01-01

**Authors:** Jiyeong Song, Young-A Lee, Dongwon Kim

**Affiliations:** a Department of Chemistry, Jeonbuk National University, Jeonju 54896, Republic of Korea; b Beamline Department, Pohang Acceleratory Laboratory, Pohang 37673, Republic of Korea; University of Aberdeen, United Kingdom

**Keywords:** crystal structure, diphenyl-4-pyridyl­phosphine ligand, silver(I), coordination polymer, tri­fluoro­acetate

## Abstract

The crystal structure of the title compound, [Ag(CF_3_CO_2_)(C_17_H_14_NP)] has been determined from synchrotron data (λ = 0.70000 Å). Centrosymmetric dinuclear Ag_2_O_2_ units, generated by inversion centers, extend into a two-dimensional coordination polymer linked by the N and P atoms of the bridged-bidentate (μ^2^) ligand. The resulting two-dimensional array can be described as a 6^3^ (hcb) network.

## Abstract 2

In the title coordination polymer, μ_2_-*N*,*P*-bridging diphen­yl(4-pyrid­yl)phosphane and O-bonded tri­fluoro­acetate ligands generate centrosymmetric dinuclear Ag^I^ units that extend into a two-dimensional hcb coordination network.

## 1. Chemical context

Research on the construction and packing motifs of two-dimensional coordination networks is of inter­est due to their many applications such as adsorption/desorption, mol­ecular recognition, separation, energy transfer template, chemo-sensors, ion exchangers, drying agents, nuclear waste storage, crystal structure templates of liquids and heterogeneous catalysis (Kim *et al.*, 2022 ▸) *via* the stretching and sliding between layers (Kim *et al.*, 2021 ▸). Thus, new types of two-dimensional coordination frameworks have been constructed by the self-assembly of metal cations as a geometric component and designed multidonor ligands as a spacer. Furthermore, such frameworks could be rationally designed by controlling weakly coordinating (counter)anions due to the less effective electrostatic binding inter­actions. Anion chemistry has emerged as an active field owing to a timely inter­est from environmental pollution, industrial chemicals, biological process, ionic liquids, catalysis, lithium battery, and health-related perspectives (Beer & Gale, 2001 ▸). Among ligands, diphenyl-4-pyridyl phosphine spacers exhibit delicate differences in the size, lone-pair delocalization, conformational energy barrier, and donating ability (Wang *et al.*, 2004 ▸). Here, we describe the synthesis and crystal structure of the title coordination polymer [Ag(CF_3_CO_2_)(C_17_H_14_NP)]_*n*_, (**I**).
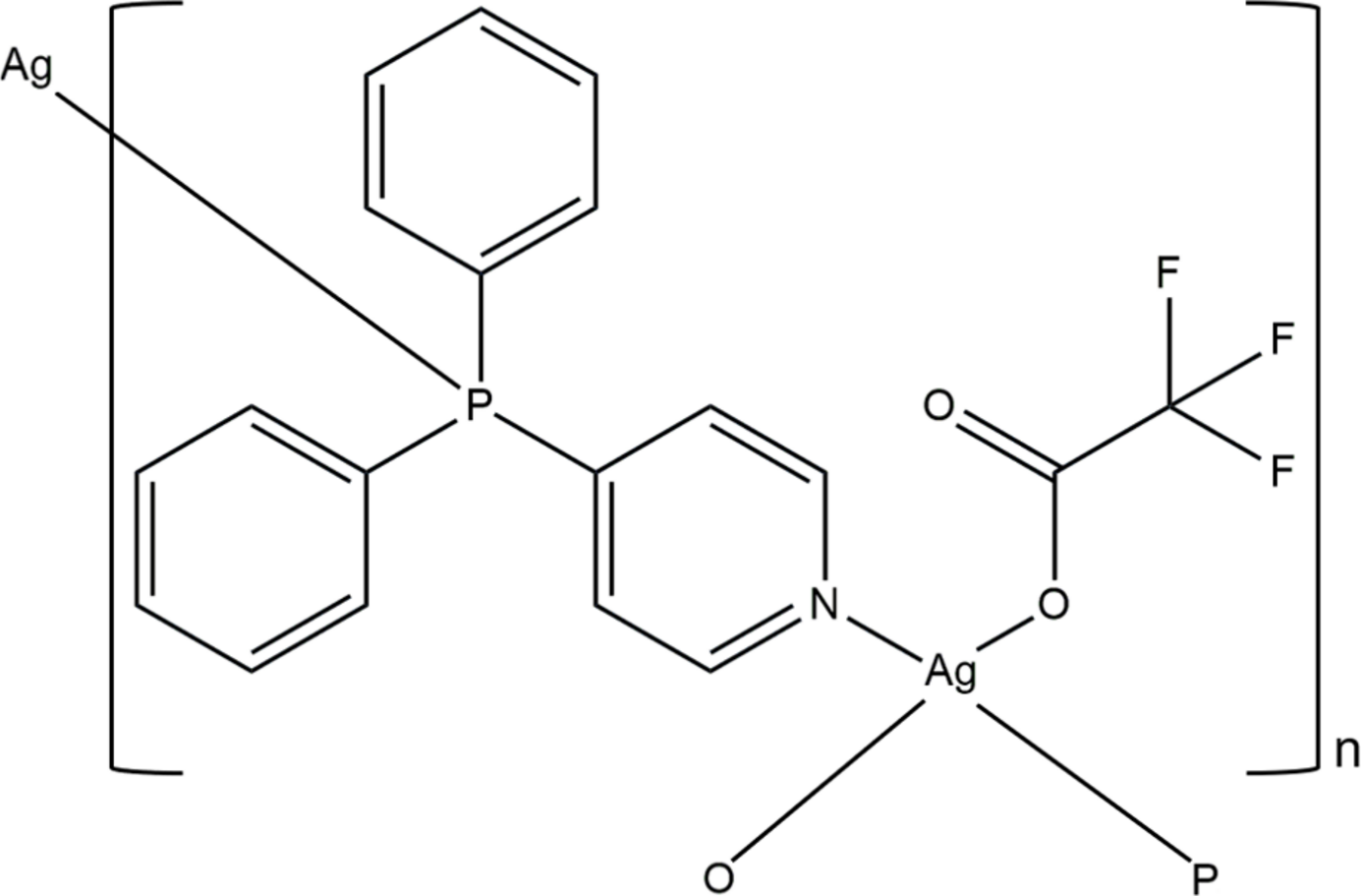


## 2. Structural commentary

**Figure 1:**
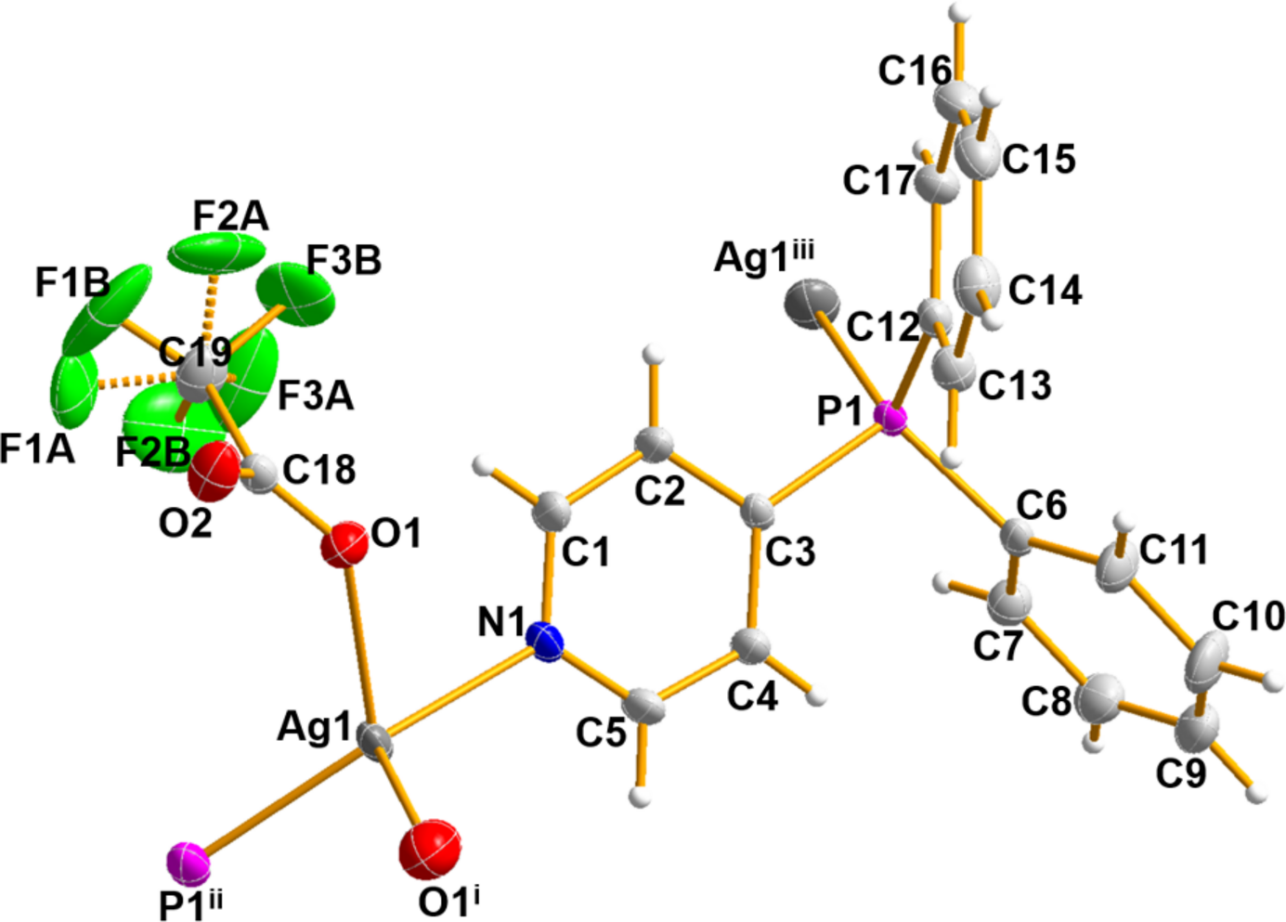
The asymmetric unit of (**I**) expanded to show the complete silver-ion coordination sphere with displacement ellipsoids drawn at the 30% probability level. [Symmetry codes: (i) −*x* + 1, −*y* + 2, −*z* + 1; (ii) −*x* + 1, *y* + 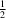, −*z* + 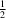; (iii) −*x* + 1, *y* − 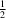, −*z* + 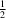.]

The metal ion in (**I**) is coordinated by the N and P atoms of two diphenyl-4-pyridyl phosphine (*L*) ligands (one symmetry generated) and two O-atom donors of the bridging tri­fluoro­acetate anions (one symmetry generated) and selected bond lengths and angles are listed in Table 1 ▸. This results in a very distorted AgO_2_NP tetra­hedral arrangement (Fig. 1 ▸) with the N—Ag—P bond angle being 142.00 (6)°. The O atoms bridge to an adjacent silver atom, which thus forms a centrosymmetric dinuclear unit generated by an inversion center at (1/2, 1, 1/2) for the asymmetric atoms. A short Ag1⋯O2(1 − *x*, 2 − *y*, 1 − *z*) contact of 2.764 (2) Å arises within the dimer.

**Figure 2:**
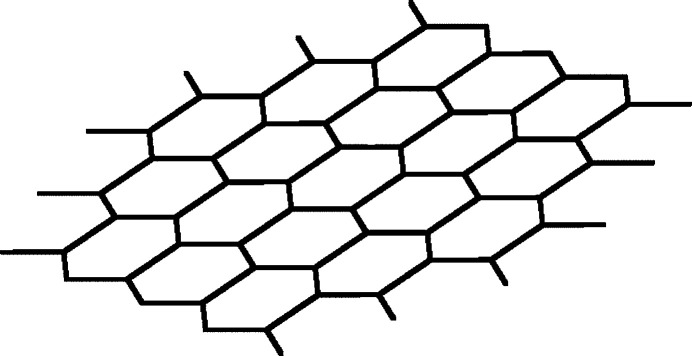
Schematic representation of the hcb (6^3^) topology of title compound. Topological analysis was performed with a metal-centered simplification, treating the central point between the Ag_2_ pairs as nodes and the diphen­yl(4-pyrid­yl)phosphane ligands as linkers.

**Figure 3:**
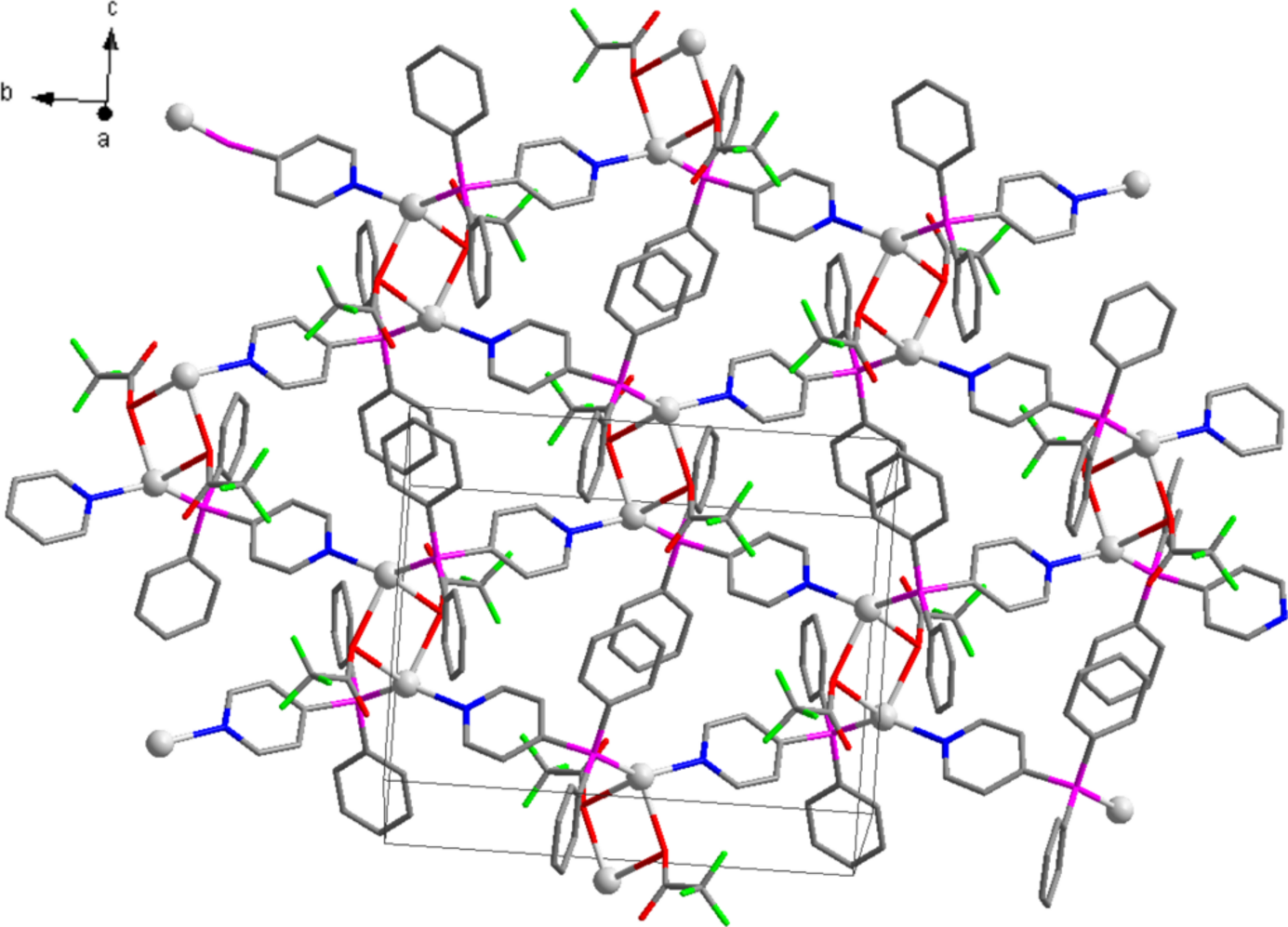
The two-dimensional network structure of title compound. For clarity, H atoms have been omitted.

The C_17_H_14_NP (*L*) spacer ligand connects two silver(I) ions to give a single polymeric strand propagating in the [010] direction. The two O-atom donors of the tri­fluoro­acetate anion bridge the single strands in an ‘up and down’ mode and the resulting extended structure is a two-dimensional coordination polymer propagating in the (100) plane, as shown in Fig. 2 ▸, with an hcb 6^3^ topology (O’Keeffe *et al.*, 2008 ▸). The double-bridge *via* the two acetate O atoms induces an Ag⋯Ag(1 − *x*, 2 − *y*, 1 − *z*) distance of 3.7495 (8) Å, which is probably too long to be regarded as an argentophilic (Pyykkö, 1997 ▸) silver–silver ‘bond’. A weak C—H⋯O inter­action (Table 2 ▸, Fig. 3 ▸) may help to consolidate the sheets. No directional inter­actions could be identified in the inter-layer packing.

## 3. Database survey

A search of the Cambridge Structural Database (CSD, version 6.00 with updates through April 2025; Groom *et al.*, 2016 ▸) using ConQuest was made for metal complexes containing diphen­yl(4-pyrid­yl)phosphane ligands. Among the 18 hits retrieved, no closely related structure to the title complex was found.

## 4. Synthesis and crystallization

A solution was prepared by dissolving AgCF_3_CO_2_ (4.42 mg, 0.020 mmol) in acetone, and another solution was prepared by dissolving diphenyl-4-pyridyl phosphine (5.27 mg, 0.020 mmol) in ethanol. Slow diffusion of the two solutions over several days afforded colorless block-shaped crystals of (**I**) suitable for X-ray diffraction. Yield: 8.33 mg (86%).

## 5. Refinement

Crystal data, data collection and structure refinement details are summarized in Table 3 ▸. All H atoms were placed in geometrically idealized positions and constrained to ride on their parent atoms, with C—H = 0.94 Å and *U*_iso_(H) = 1.2 *U*_eq_(carrier). The F atoms of the –CF_3_ group are disordered over two sets of sites in a 0.510 (13):0.490 (13) ratio.

## Supplementary Material

Crystal structure: contains datablock(s) I. DOI: 10.1107/S2056989025011302/hb8176sup1.cif

Structure factors: contains datablock(s) I. DOI: 10.1107/S2056989025011302/hb8176Isup2.hkl

CSD search file. DOI: 10.1107/S2056989025011302/hb8176sup3.pdf

CCDC reference: 2515821

Additional supporting information:  crystallographic information; 3D view; checkCIF report

## Figures and Tables

**Table 1:** Selected geometric parameters (Å, °)

Ag1—N1	2.242 (2)	Ag1—O1	2.463 (2)
Ag1—P1^i^	2.3594 (7)	Ag1—O1^ii^	2.588 (2)
			
N1—Ag1—P1^i^	142.00 (6)	N1—Ag1—O1^ii^	113.05 (7)
N1—Ag1—O1	87.13 (8)	P1^i^—Ag1—O1^ii^	98.45 (5)
P1^i^—Ag1—O1	117.84 (6)	O1—Ag1—O1^ii^	84.18 (7)

Symmetry codes: (i) 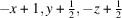; (ii) 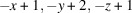.

**Table 2:** Hydrogen-bond geometry (Å, °)

*D*—H⋯*A*	*D*—H	H⋯*A*	*D*⋯*A*	*D*—H⋯*A*
C1—H1⋯O1	0.94	2.53	3.160 (3)	124

**Table 3:** Experimental details

Crystal data	
Chemical formula	[Ag(C_2_F_3_O_2_)(C_17_H_14_NP)]
*M*_r_	484.15
Crystal system, space group	Monoclinic, *P*2_1_/*c*
Temperature (K)	223
*a*, *b*, *c* (Å)	11.683 (2), 14.843 (3), 11.335 (2)
β (°)	103.68 (3)
*V* (Å^3^)	1909.8 (7)
*Z*	4
Radiation type	Synchrotron, λ = 0.700 Å
μ (mm^−1^)	1.12
Crystal size (mm)	0.30 × 0.22 × 0.12

Data collection	
Diffractometer	Rayonix MX225HS CCD area detector
Absorption correction	Multi-scan (*HKL3000sm *SCALEPACK**; Otwinowski *et al.*, 2003 ▸)
*T*_min_, *T*_max_	0.939, 1.000
No. of measured, independent and observed [*I* > 2σ(*I*)] reflections	19907, 5327, 4578
*R*_int_	0.042
(sin θ/λ)_max_ (Å^−1^)	0.704

Refinement	
*R*[*F*^2^ > 2σ(*F*^2^)], *wR*(*F*^2^), *S*	0.043, 0.116, 1.11
No. of reflections	5327
No. of parameters	272
No. of restraints	36
H-atom treatment	H-atom parameters constrained
Δρ_max_, Δρ_min_ (e Å^−3^)	0.40, −1.86

Computer programs: *PAL BL2D-SMDC Program* (Shin *et al.*, 2016 ▸), *HKL3000sm* (Otwinowski & Minor, 2003 ▸), *SHELXT2018* (Sheldrick, 2015*a* ▸), *SHELXL2018* (Sheldrick, 2015*b* ▸), *DIAMOND* (Putz & Brandenburg, 2014 ▸) and *publCIF* (Westrip, 2010 ▸).

## supplementary crystallographic information

### Poly[µ-diphenyl(pyridin-4-yl)phosphane-κ2N:P-µ-trifluoroacetato-κ2O:O'-silver(I)] Crystal data

**Table:**

[Ag(C_2_F_3_O_2_)(C_17_H_14_NP)]	*F*(000) = 960
*M**_r_* = 484.15	*D*_x_ = 1.684 Mg m^−^^3^
Monoclinic, *P*2_1_/*c*	Synchrotron radiation, λ = 0.700 Å
*a* = 11.683 (2) Å	Cell parameters from 40378 reflections
*b* = 14.843 (3) Å	θ = 0.4–29.5°
*c* = 11.335 (2) Å	µ = 1.12 mm^−^^1^
β = 103.68 (3)°	*T* = 223 K
*V* = 1909.8 (7) Å^3^	Block, colorless
*Z* = 4	0.30 × 0.22 × 0.12 mm

### Poly[µ-diphenyl(pyridin-4-yl)phosphane-κ2N:P-µ-trifluoroacetato-κ2O:O'-silver(I)] Data collection

**Table:**

Rayonix MX225HS CCD area detector diffractometer	4578 reflections with *I* > 2σ(*I*)
Radiation source: PLSII 2D bending magnet	*R*_int_ = 0.042
ω scan	θ_max_ = 29.5°, θ_min_ = 2.2°
Absorption correction: multi-scan (*HKL3000sm Scalepack*; Otwinowski *et al.*, 2003)	*h* = −16→16
*T*_min_ = 0.939, *T*_max_ = 1.000	*k* = −20→20
19907 measured reflections	*l* = −14→14
5327 independent reflections	

### Poly[µ-diphenyl(pyridin-4-yl)phosphane-κ2N:P-µ-trifluoroacetato-κ2O:O'-silver(I)] Refinement

**Table:**

Refinement on *F*^2^	Primary atom site location: dual
Least-squares matrix: full	Hydrogen site location: inferred from neighbouring sites
*R*[*F*^2^ > 2σ(*F*^2^)] = 0.043	H-atom parameters constrained
*wR*(*F*^2^) = 0.116	*w* = 1/[σ^2^(*F*_o_^2^) + (0.0728*P*)^2^ + 0.3483*P*] where *P* = (*F*_o_^2^ + 2*F*_c_^2^)/3
*S* = 1.11	(Δ/σ)_max_ = 0.001
5327 reflections	Δρ_max_ = 0.40 e Å^−^^3^
272 parameters	Δρ_min_ = −1.86 e Å^−^^3^
36 restraints	

### Poly[µ-diphenyl(pyridin-4-yl)phosphane-κ2N:P-µ-trifluoroacetato-κ2O:O'-silver(I)] Special details

**Table:**

Geometry. All esds (except the esd in the dihedral angle between two l.s. planes) are estimated using the full covariance matrix. The cell esds are taken into account individually in the estimation of esds in distances, angles and torsion angles; correlations between esds in cell parameters are only used when they are defined by crystal symmetry. An approximate (isotropic) treatment of cell esds is used for estimating esds involving l.s. planes.

### Poly[µ-diphenyl(pyridin-4-yl)phosphane-κ2N:P-µ-trifluoroacetato-κ2O:O'-silver(I)] Fractional atomic coordinates and isotropic or equivalent isotropic displacement parameters (Å^2^)

**Table:**

	*x*	*y*	*z*	*U*_iso_*/*U*_eq_	Occ. (<1)
C3	0.35805 (18)	0.68394 (14)	0.2085 (2)	0.0271 (4)	
C4	0.3393 (2)	0.76130 (16)	0.1382 (2)	0.0355 (5)	
H4	0.288044	0.760537	0.060553	0.043*	
C5	0.3975 (2)	0.83998 (17)	0.1840 (2)	0.0391 (5)	
H5	0.383473	0.892220	0.135841	0.047*	
C6	0.17431 (19)	0.60043 (15)	0.0265 (2)	0.0299 (4)	
C7	0.2086 (2)	0.6029 (2)	−0.0828 (2)	0.0411 (5)	
H7	0.287002	0.589939	−0.084168	0.049*	
C8	0.1274 (3)	0.6243 (2)	−0.1901 (3)	0.0525 (7)	
H8	0.151235	0.626321	−0.263645	0.063*	
C9	0.0125 (3)	0.6427 (2)	−0.1886 (3)	0.0567 (8)	
H9	−0.042092	0.657774	−0.260991	0.068*	
C10	−0.0227 (3)	0.6389 (3)	−0.0807 (3)	0.0659 (10)	
H10	−0.101556	0.650869	−0.080129	0.079*	
C11	0.0577 (2)	0.6176 (2)	0.0269 (3)	0.0487 (7)	
H11	0.033058	0.614747	0.099967	0.058*	
C12	0.2249 (2)	0.54983 (16)	0.2876 (2)	0.0315 (4)	
C13	0.1573 (2)	0.61216 (19)	0.3340 (2)	0.0404 (5)	
H13	0.138279	0.668030	0.295284	0.048*	
C14	0.1185 (3)	0.5915 (2)	0.4371 (3)	0.0511 (7)	
H14	0.071082	0.632748	0.466804	0.061*	
C15	0.1493 (3)	0.5101 (3)	0.4971 (3)	0.0549 (8)	
H15	0.124504	0.497203	0.568348	0.066*	
C16	0.2158 (3)	0.4488 (2)	0.4520 (3)	0.0525 (7)	
H16	0.235659	0.393394	0.491779	0.063*	
C17	0.2539 (3)	0.46832 (17)	0.3475 (3)	0.0402 (6)	
H17	0.299643	0.426076	0.317180	0.048*	
C18	0.6472 (2)	0.90684 (16)	0.6714 (2)	0.0352 (5)	
C19	0.7446 (3)	0.8351 (2)	0.6859 (3)	0.0579 (8)	
Ag1	0.56236 (2)	0.97380 (2)	0.36594 (2)	0.03465 (8)	
P1	0.28994 (5)	0.57488 (4)	0.16075 (5)	0.02680 (12)	
O1	0.57810 (19)	0.91102 (15)	0.57033 (18)	0.0479 (5)	
N1	0.47206 (18)	0.84526 (14)	0.29276 (19)	0.0352 (4)	
C1	0.4910 (2)	0.76980 (18)	0.3596 (2)	0.0418 (6)	
H1	0.543413	0.772179	0.436476	0.050*	
O2	0.6465 (2)	0.95088 (17)	0.7623 (2)	0.0584 (6)	
C2	0.4374 (2)	0.68904 (17)	0.3208 (2)	0.0402 (6)	
H2	0.454302	0.637557	0.370122	0.048*	
F1A	0.8505 (5)	0.8723 (6)	0.7220 (11)	0.098 (3)	0.490 (13)
F2A	0.7521 (10)	0.7926 (10)	0.5923 (11)	0.131 (6)	0.490 (13)
F3A	0.7391 (10)	0.7777 (8)	0.7754 (14)	0.129 (5)	0.490 (13)
F1B	0.8208 (12)	0.8362 (9)	0.7831 (10)	0.152 (6)	0.510 (13)
F2B	0.7951 (11)	0.8384 (10)	0.5961 (13)	0.148 (6)	0.510 (13)
F3B	0.6952 (7)	0.7545 (3)	0.6669 (13)	0.108 (4)	0.510 (13)

### Poly[µ-diphenyl(pyridin-4-yl)phosphane-κ2N:P-µ-trifluoroacetato-κ2O:O'-silver(I)] Atomic displacement parameters (Å^2^)

**Table:**

	*U*^11^	*U*^22^	*U*^33^	*U*^12^	*U*^13^	*U*^23^
C3	0.0229 (9)	0.0185 (9)	0.0390 (10)	0.0001 (7)	0.0055 (7)	−0.0020 (7)
C4	0.0365 (12)	0.0235 (11)	0.0417 (12)	−0.0019 (9)	−0.0003 (9)	0.0029 (9)
C5	0.0459 (14)	0.0225 (11)	0.0447 (13)	−0.0042 (10)	0.0027 (10)	0.0045 (9)
C6	0.0252 (10)	0.0194 (10)	0.0420 (11)	−0.0016 (7)	0.0018 (8)	−0.0020 (8)
C7	0.0388 (13)	0.0396 (14)	0.0434 (13)	0.0016 (11)	0.0068 (10)	−0.0017 (10)
C8	0.0578 (18)	0.0528 (19)	0.0421 (15)	−0.0041 (15)	0.0022 (12)	−0.0001 (12)
C9	0.0498 (17)	0.0537 (19)	0.0544 (16)	−0.0058 (14)	−0.0120 (13)	0.0035 (14)
C10	0.0270 (13)	0.091 (3)	0.072 (2)	0.0048 (15)	−0.0051 (13)	0.0061 (19)
C11	0.0266 (12)	0.064 (2)	0.0531 (15)	0.0044 (12)	0.0051 (10)	0.0049 (13)
C12	0.0280 (10)	0.0255 (10)	0.0399 (12)	−0.0044 (8)	0.0060 (8)	−0.0012 (8)
C13	0.0358 (12)	0.0356 (13)	0.0508 (14)	0.0005 (10)	0.0123 (10)	−0.0028 (10)
C14	0.0417 (15)	0.060 (2)	0.0552 (17)	−0.0048 (13)	0.0178 (12)	−0.0128 (14)
C15	0.0527 (18)	0.069 (2)	0.0431 (16)	−0.0190 (16)	0.0112 (12)	0.0012 (14)
C16	0.0583 (19)	0.0473 (17)	0.0491 (16)	−0.0135 (15)	0.0070 (13)	0.0109 (13)
C17	0.0433 (15)	0.0316 (13)	0.0442 (14)	−0.0018 (10)	0.0074 (11)	0.0046 (9)
C18	0.0306 (11)	0.0244 (11)	0.0487 (13)	−0.0007 (8)	0.0057 (9)	0.0011 (9)
C19	0.0427 (16)	0.0526 (19)	0.074 (2)	0.0143 (14)	0.0052 (14)	−0.0084 (16)
Ag1	0.03016 (12)	0.02218 (11)	0.05095 (14)	−0.00674 (6)	0.00828 (8)	−0.00112 (6)
P1	0.0226 (3)	0.0167 (2)	0.0394 (3)	−0.00019 (18)	0.0039 (2)	−0.00062 (19)
O1	0.0444 (11)	0.0526 (13)	0.0439 (10)	0.0006 (9)	0.0047 (8)	0.0074 (9)
N1	0.0329 (10)	0.0222 (9)	0.0482 (11)	−0.0044 (7)	0.0049 (8)	−0.0011 (8)
C1	0.0403 (13)	0.0287 (12)	0.0480 (13)	−0.0061 (10)	−0.0063 (10)	0.0012 (10)
O2	0.0557 (14)	0.0507 (13)	0.0622 (14)	0.0094 (11)	0.0011 (10)	−0.0211 (11)
C2	0.0399 (13)	0.0248 (11)	0.0470 (13)	−0.0042 (10)	−0.0075 (10)	0.0056 (9)
F1A	0.026 (2)	0.112 (6)	0.148 (8)	0.003 (3)	0.006 (3)	−0.011 (5)
F2A	0.091 (6)	0.148 (9)	0.134 (8)	0.060 (6)	−0.009 (5)	−0.092 (7)
F3A	0.107 (6)	0.100 (7)	0.194 (11)	0.066 (5)	0.063 (7)	0.099 (7)
F1B	0.135 (9)	0.154 (10)	0.115 (7)	0.104 (8)	−0.074 (6)	−0.052 (6)
F2B	0.121 (8)	0.169 (10)	0.199 (11)	0.054 (7)	0.124 (8)	0.026 (8)
F3B	0.102 (5)	0.028 (2)	0.194 (10)	0.025 (3)	0.035 (6)	0.003 (4)

### Poly[µ-diphenyl(pyridin-4-yl)phosphane-κ2N:P-µ-trifluoroacetato-κ2O:O'-silver(I)] Geometric parameters (Å, º)

**Table:**

C3—C4	1.385 (3)	C15—C16	1.372 (6)
C3—C2	1.388 (3)	C16—C17	1.391 (4)
C3—P1	1.828 (2)	C18—O2	1.222 (3)
C4—C5	1.389 (3)	C18—O1	1.238 (3)
C5—N1	1.333 (3)	C18—C19	1.538 (4)
C6—C11	1.387 (3)	C19—F1B	1.242 (7)
C6—C7	1.390 (3)	C19—F2A	1.255 (8)
C6—P1	1.820 (2)	C19—F2B	1.292 (9)
C7—C8	1.391 (4)	C19—F3B	1.325 (7)
C8—C9	1.374 (5)	C19—F1A	1.327 (7)
C9—C10	1.380 (5)	C19—F3A	1.339 (8)
C10—C11	1.389 (4)	Ag1—N1	2.242 (2)
C12—C17	1.390 (3)	Ag1—P1^i^	2.3594 (7)
C12—C13	1.397 (4)	Ag1—O1	2.463 (2)
C12—P1	1.816 (2)	Ag1—O1^ii^	2.588 (2)
C13—C14	1.384 (4)	N1—C1	1.341 (3)
C14—C15	1.391 (5)	C1—C2	1.376 (3)
			
C4—C3—C2	117.6 (2)	F2A—C19—F3A	110.2 (8)
C4—C3—P1	124.43 (17)	F1A—C19—F3A	103.9 (6)
C2—C3—P1	117.97 (17)	F1B—C19—C18	116.3 (5)
C3—C4—C5	119.0 (2)	F2A—C19—C18	117.1 (5)
N1—C5—C4	123.4 (2)	F2B—C19—C18	110.7 (6)
C11—C6—C7	119.2 (2)	F3B—C19—C18	108.9 (4)
C11—C6—P1	124.9 (2)	F1A—C19—C18	110.9 (5)
C7—C6—P1	115.93 (18)	F3A—C19—C18	110.8 (4)
C6—C7—C8	120.3 (3)	N1—Ag1—P1^i^	142.00 (6)
C9—C8—C7	120.0 (3)	N1—Ag1—O1	87.13 (8)
C8—C9—C10	120.0 (3)	P1^i^—Ag1—O1	117.84 (6)
C9—C10—C11	120.4 (3)	N1—Ag1—O1^ii^	113.05 (7)
C6—C11—C10	120.0 (3)	P1^i^—Ag1—O1^ii^	98.45 (5)
C17—C12—C13	119.1 (2)	O1—Ag1—O1^ii^	84.18 (7)
C17—C12—P1	117.8 (2)	C12—P1—C6	109.72 (11)
C13—C12—P1	122.8 (2)	C12—P1—C3	100.46 (10)
C14—C13—C12	119.9 (3)	C6—P1—C3	104.35 (10)
C13—C14—C15	120.5 (3)	C12—P1—Ag1^iii^	115.27 (8)
C16—C15—C14	119.9 (3)	C6—P1—Ag1^iii^	116.50 (8)
C15—C16—C17	120.2 (3)	C3—P1—Ag1^iii^	108.70 (7)
C12—C17—C16	120.5 (3)	C18—O1—Ag1	140.99 (19)
O2—C18—O1	128.4 (3)	C18—O1—Ag1^ii^	95.32 (17)
O2—C18—C19	115.6 (3)	Ag1—O1—Ag1^ii^	95.82 (7)
O1—C18—C19	116.0 (2)	C5—N1—C1	117.2 (2)
F1B—C19—F2B	109.5 (9)	C5—N1—Ag1	122.66 (17)
F1B—C19—F3B	110.4 (7)	C1—N1—Ag1	120.10 (16)
F2B—C19—F3B	99.7 (7)	N1—C1—C2	123.1 (2)
F2A—C19—F1A	102.9 (7)	C1—C2—C3	119.7 (2)

Symmetry codes: (i) −*x*+1, *y*+1/2, −*z*+1/2; (ii) −*x*+1, −*y*+2, −*z*+1; (iii) −*x*+1, *y*−1/2, −*z*+1/2.

### Poly[µ-diphenyl(pyridin-4-yl)phosphane-κ2N:P-µ-trifluoroacetato-κ2O:O'-silver(I)] Hydrogen-bond geometry (Å, º)

**Table:**

*D*—H···*A*	*D*—H	H···*A*	*D*···*A*	*D*—H···*A*
C1—H1···O1	0.94	2.53	3.160 (3)	124
