# Supplementary material for: Crystal structure of poly[μ-diphen­yl(pyridin-4-yl)phosphane-κ2N:P-μ-tri­fluoro­acetato-κ2O:O′-silver(I)] from synchrotron data
Source: Acta Crystallogr E Crystallogr Commun. 2026 Jan 1;82(Pt 1):96–8. doi: 10.1107/S2056989025011302 (PMC12810293; doi:10.1107/S2056989025011302)

Search Overview

Search: search1

Date/Time done: Tue Nov 25 10:01:16 2025

Database(s): CSD version 6.00 (Apr 2025)

Restriction Info: No refcode restrictions applied

Filters: None

Percentage Completed: 100%

Number of Hits: 18

Single query used. Search found structures that:

match

Query 1

Query 1

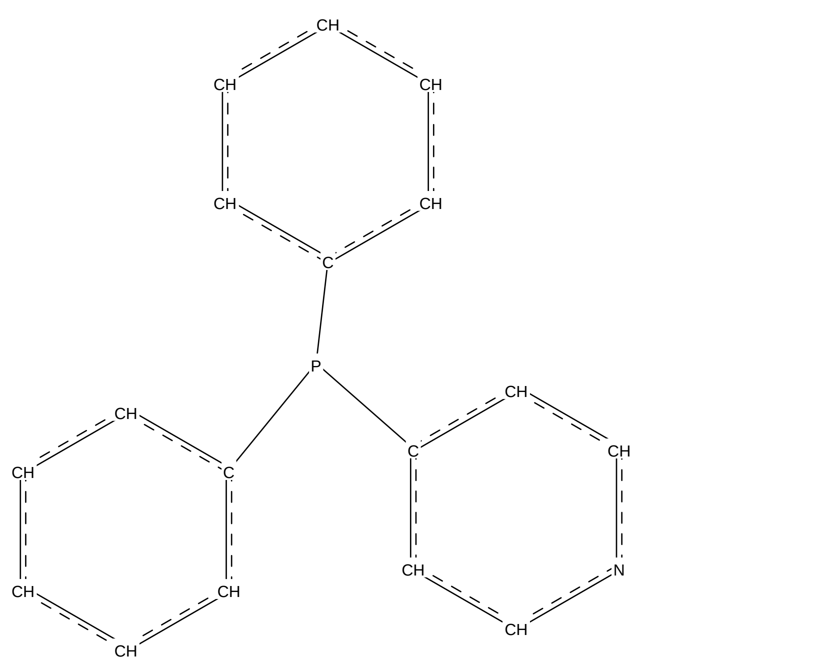

Search: search1 (Tue Nov 25 10:01:16 2025): Hits 1-4

ALAMIK

Reference: I.Angurell, M.Ferrer, A.Gutierrez, M.Martinez, L.Rodriguez, O.Rossell, M.Engenser (2010) *Chem.-Eur.J.* ,**16,**13960

Formula: $C_{130} H_{122} N_4 P_8 Pd_2 Pt_2{}^{6+}$,$C_4 H_{10} O_1$,6($C_1 F_3 O_3 S_1{}^{1-}$),2.67($H_2 O_1$),2($C_1 H_2 Cl_2$)

Compound Name: tetrakis(μ_2-Diphenyl(4-pyridyl)phosphine)-bis(1,3-bis(diphenylphosphino) propane)-bis(η^3-2-methylallyl)-di-palladium-di-platinum hexakis(trifluoromethanesulfonate) dichloromethane diethyl ether solvate hydrate

Space Group:	P-1	***Cell:***	***a*** 14.139(0)	***b*** 32.308(1)	***c*** 52.607(3)
Space Group No.:	2	***(Å,°)***	α 94.87(0)	β 90.93(0)	γ 93.91(0)

R-Factor (%): 7.81 ***Temperature(K):*** 100 ***Density(g/cm³):*** 1.576

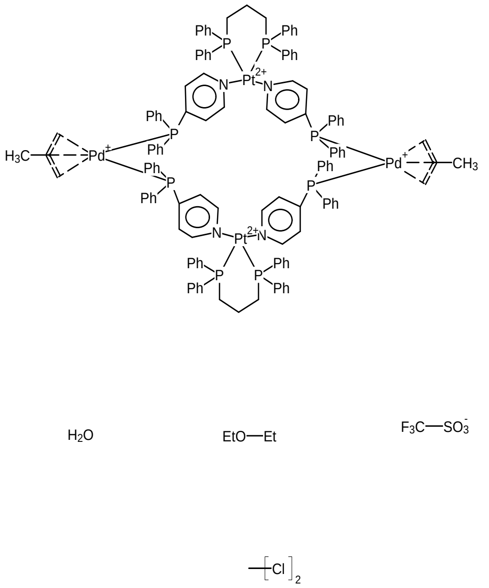

COSYIT

Reference: A.M.Kluwer, R.Kapre, F.Hartl, M.Lutz, A.L.Spek, A.M.Brouwer, P.W.N.M.van Leeuwen, J.N.H.Reek (2009) *Proc.Nat.Acad.Sci.USA* ,**106,**10460

Formula: $C_{25} H_{20} Fe_2 N_1 O_5 P_1 S_2$

Compound Name: (μ_2-1,3-Propanedithiolato-1κ^2S^1,S^3:2κ^2S^1,S^3)-pentacarbonyl-(4-(diphenylphosphino-2κP)pyridine)-di-iron

Space Group:	P-1	***Cell:***	***a*** 9.300(0)	***b*** 10.092(0)	***c*** 14.711(0)
Space Group No.:	2	***(Å,°)***	α 94.83(0)	β 97.04(0)	γ 108.49(0)

R-Factor (%): 3.41 ***Temperature(K):*** 150 ***Density(g/cm³):*** 1.601

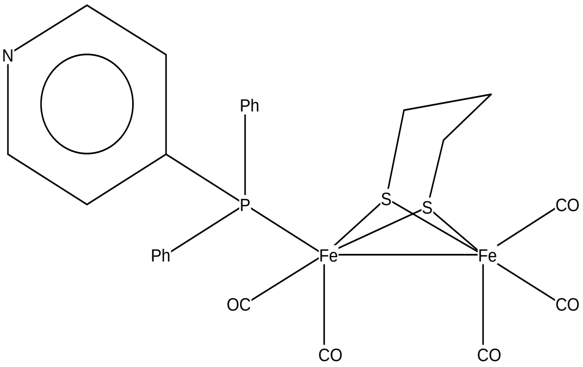

COSYOZ

Reference: A.M.Kluwer, R.Kapre, F.Hartl, M.Lutz, A.L.Spek, A.M.Brouwer, P.W.N.M.van Leeuwen, J.N.H.Reek (2009) *Proc.Nat.Acad.Sci.USA* ,**106,**10460

Formula: $C_{41} H_{34} Fe_2 N_2 O_4 P_2 S_2$,0.8($C_1 H_2 Cl_2$)

Compound Name: (μ_2-1,3-Propanedithiolato-1κ^2S^1,S^3:2κ^2S^1,S^3)-tetracarbonyl-bis(4-(diphenylphosphino-2κP)pyridine)-di-iron dichloromethane solvate

Space Group:	P21/c	***Cell:***	***a*** 9.023(0)	***b*** 22.686(0)	***c*** 21.114(0)
Space Group No.:	14	***(Å,°)***	α 90.00	β 95.07(0)	γ 90.00

R-Factor (%): 4.09 ***Temperature(K):*** 125 ***Density(g/cm³):*** 1.426

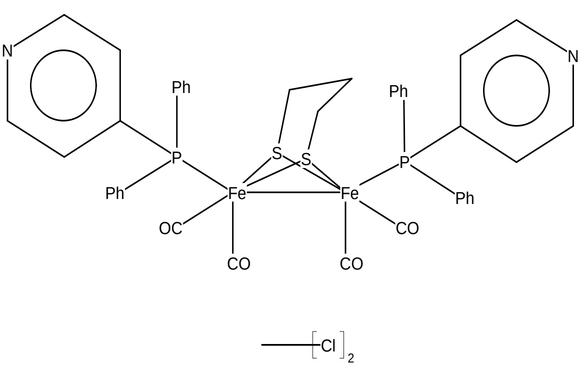

COSZAM

Reference: A.M.Kluwer, R.Kapre, F.Hartl, M.Lutz, A.L.Spek, A.M.Brouwer, P.W.N.M.van Leeuwen, J.N.H.Reek (2009) *Proc.Nat.Acad.Sci.USA* ,**106,**10460

Formula: $C_{69} H_{48} Fe_2 N_5 O_5 P_1 S_2 Zn_1$,2($C_1 H_2 Cl_2$)

Compound Name: (μ_2-4-(Diphenylphosphino-2κP)pyridine-N)-(μ_2-1,3-propanedithiolato-1κ^2S^1,S^3:2κ^2S^1,S^3)-pentacarbonyl-(5,10,15,20-tetraphenylporphyrinato) -di-iron-zinc(ii) dichloromethane solvate

Space Group:	P-1	***Cell:***	***a*** 11.147(0)	***b*** 28.182(0)	***c*** 32.849(0)
Space Group No.:	2	***(Å,°)***	α 75.41(0)	β 83.95(0)	γ 78.94(0)

R-Factor (%): 6.71 ***Temperature(K):*** 150 ***Density(g/cm³):*** 1.496

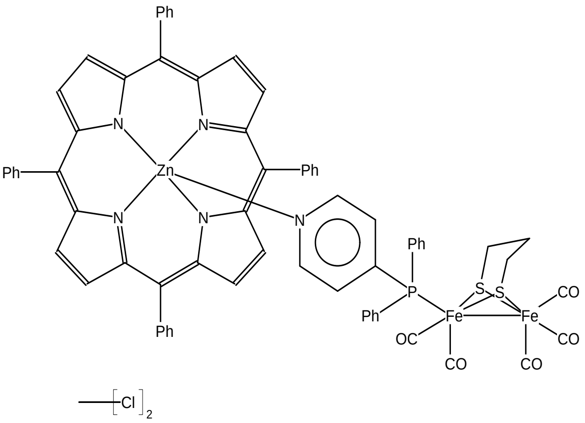

Search: search1 (Tue Nov 25 10:01:16 2025): Hits 5-8

DAQJAI

Reference: V.Bocokic, M.Lutz, A.L.Spek, J.N.H.Reek (2012) *Dalton Trans.* ,**41,**3740

Formula: $C_{31} H_{32} N_7 P_1 S_2 Zn_1, C_7 H_8$

Compound Name: (N'-(3-((Anilino(sulfanyl)methylene)hydrazono)butan-2-ylidene)-N,N-dimethylcarbamohydrazonothioato)-(4-(diphenylphosphino)pyridine)-zinc(ii) toluene solvate

Space Group:	P-1	***Cell:***	***a***	12.958(0)	***b***	13.027(0)	***c***	13.141(0)
Space Group No.:	2	***(Å,°)***	α	115.99(0)	β	93.87(0)	γ	107.10(0)

R-Factor (%): 6.18 ***Temperature(K):*** 150 ***Density(g/cm^3):*** 1.352

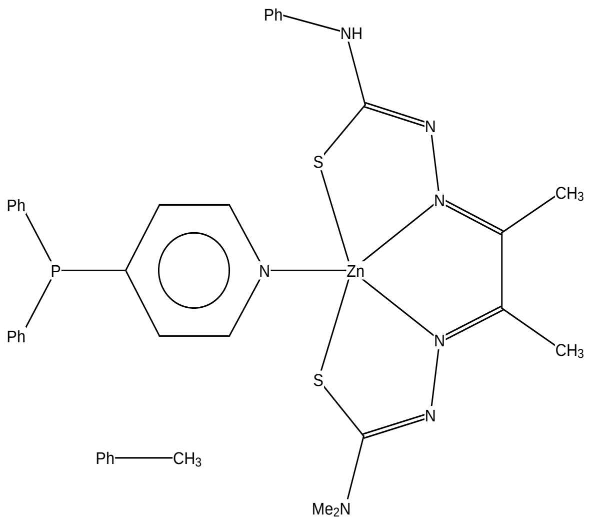

FERSAZ

Reference: A.G.Barrado, J.M.Bayne, T.C.Johnstone, C.W.Lehmann, D.W.Stephan, M.Alcarazo (2017) *Dalton Trans.* ,**46,**16216

Formula: $C_{24} H_{22} N_1 P_1{}^{2+}, 2(C_1 F_3 O_3 S_1{}^{1-})$

Compound Name: 1-methyl-4-(triphenylphosphaniumyl)pyridin-1-ium bis(trifluoromethanesulfonate)

Space Group:	P21/n	***Cell:***	***a***	9.439(0)	***b***	10.340(0)	***c***	28.977(1)
Space Group No.:	14	***(Å,°)***	α	90.00	β	94.40(0)	γ	90.00

R-Factor (%): 3.79 ***Temperature(K):*** 100 ***Density(g/cm^3):*** 1.539

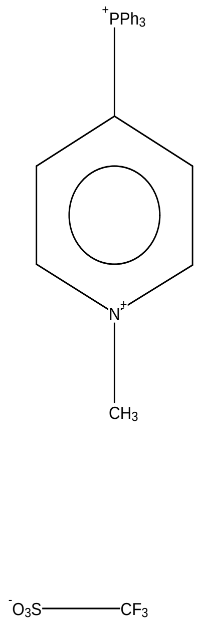

FUQHUV

Reference: A.W.Kleij, M.Lutz, A.L.Spek, J.N.H.Reek (2009) *CSD Communication(Private Communication) ,*

Formula: $C_{45} H_{42} Cl_2 N_3 O_2 P_1 Zn_1$

Compound Name: (2,2'-((4,5-Dichloro-1,2-phenylene)bis((nitrilo-κN)methylylidene))bis(6-t-butylphenolato-κO))-(4-(diphenylphosphino)pyridine-κN)-zinc(ii)

Space Group:	P21/c	***Cell:***	***a***	12.458(0)	***b***	19.835(0)	***c***	19.854(0)
Space Group No.:	14	***(Å,°)***	α	90.00	β	124.23(0)	γ	90.00

R-Factor (%): 3.01 ***Temperature(K):*** 150 ***Density(g/cm^3):*** 1.349

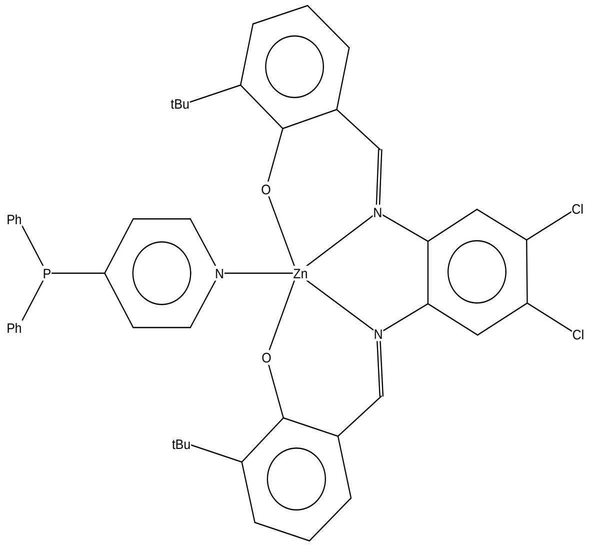

GUHLON

Reference: Shu-Peng Hu, Li Zhang, Yu Wu, Jian-Shen Feng, Sheng Wu, Bin Xie, Li-Ke Zou (2020) *Inorg.Chim.Acta* ,**504,**119435

Formula: $C_{24} H_{18} Fe_2 N_1 O_5 P_1 S_2, C_1 H_2 Cl_2$

Compound Name: [μ-ethane-1,2-dithiolato]-pentacarbonyl-[diphenyl(4-pyridyl)phosphine]-di-iron dichloromethane solvate

Space Group:	P-1	***Cell:***	***a***	8.310(2)	***b***	9.129(2)	***c***	19.215(4)
Space Group No.:	2	***(Å,°)***	α	91.81(0)	β	100.17(0)	γ	98.33(0)

R-Factor (%): 5.55 ***Temperature(K):*** 100 ***Density(g/cm^3):*** 1.622

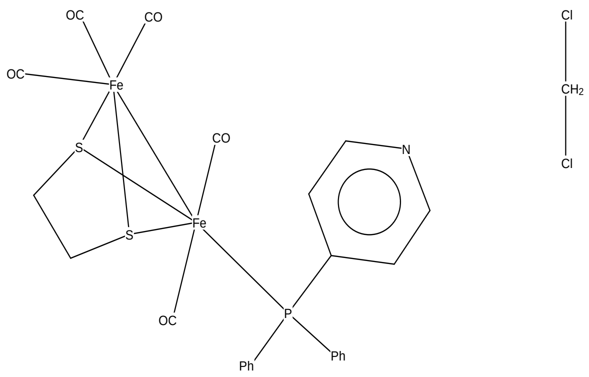

Search: search1 (Tue Nov 25 10:01:16 2025): Hits 9-12

HITLEC

Reference: C.Albrecht, S.Schwieger, T.Ruffer, C.Bruhn, T.Lis, D.Steinborn (2007) *Organometallics* ,**26,**6000

Formula: $C_{46}\ H_{52}\ Cl_2\ N_2\ O_2\ P_2\ Pt_2,2(C_1\ H_1\ Cl_3)$

Compound Name: trans-bis((μ_2-α-(Butoxy)ethylidene)-chloro-(diphenyl(4-pyridyl)phosphine))-di-platinum chloroform solvate

Space Group:	P-1	***Cell:***	***a***	9.905(1)	***b***	10.767(3)	***c***	13.881(4)
Space Group No.:	2	***(Å,°)***	α	87.27(2)	β	82.49(1)	γ	66.80(1)

R-Factor (%): 4.16 ***Temperature(K):*** 100 ***Density(g/cm^3):*** 1.756

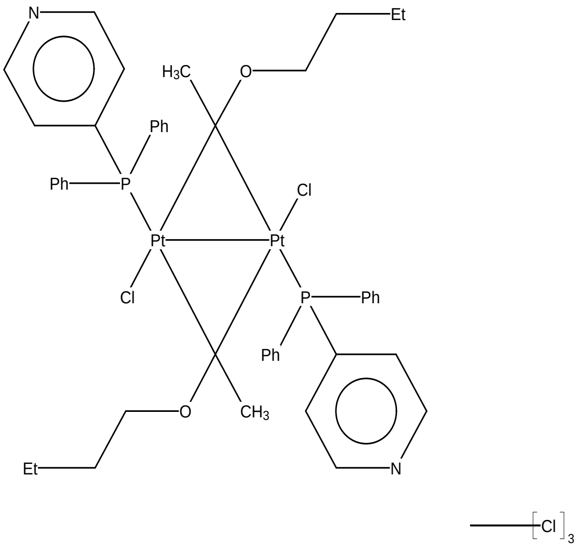

IVOYEX

Reference: E.C.Carson, S.J.Lippard (2004) *J.Am.Chem.Soc.* ,**126,** 3412

Formula: $C_{118}\ H_{96}\ Fe_2\ N_2\ O_8\ P_2,C_1\ H_2\ Cl_2$

Compound Name: tetrakis(μ_2-2,6-Di(p-tolyl)benzoato-O,O')-bis(4-(diphenylphosphino)pyridine)-di-iron(ii) dichloromethane solvate

Space Group:	C2/c	***Cell:***	***a***	22.322(5)	***b***	17.485(4)	***c***	25.357(5)
Space Group No.:	15	***(Å,°)***	α	90.00	β	105.28(0)	γ	90.00

R-Factor (%): 8.15 ***Temperature(K):*** 173 ***Density(g/cm^3):*** 1.342

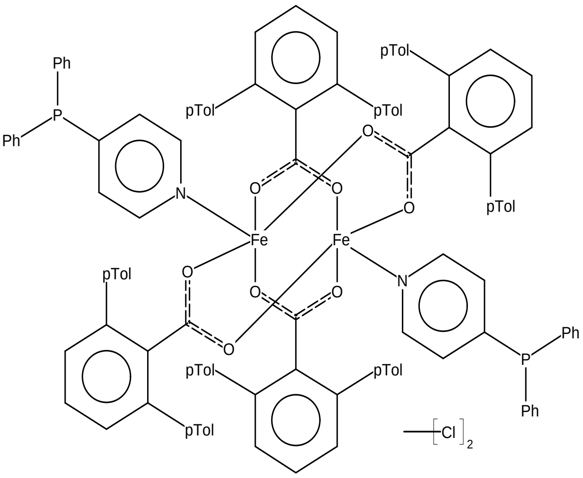

NIMXIU

Reference: M.Ferrer, A.Gallen, M.Martinez, M.Rocamora, R.Puttreddy, K.Rissanen (2022) *Dalton Trans.* ,**51,**5913

Formula: $C_{138}\ H_{134}\ N_4\ O_4\ P_8\ Pd_4\ ^{6+},6(C_1\ F_3\ O_3\ S_1\ ^{1-})$

Compound Name: tetrakis(μ-4-(diphenylphosphino)pyridine)-bis([(2,2-dimethyl-1,3-dioxolane-4,5-diyl)bis(methylene)]bis(diphenylphosphine))-bis(2-methylallyl)-tetra-palladium hexakis(trifluoromethanesulfonate) unknown solvate

Space Group:	Fddd	***Cell:***	***a***	34.089(1)	***b***	39.057(0)	***c***	53.629(2)
Space Group No.:	70	***(Å,°)***	α	90.00	β	90.00	γ	90.00

R-Factor (%): 14.43 ***Temperature(K):*** 123 ***Density(g/cm^3):*** 1.295

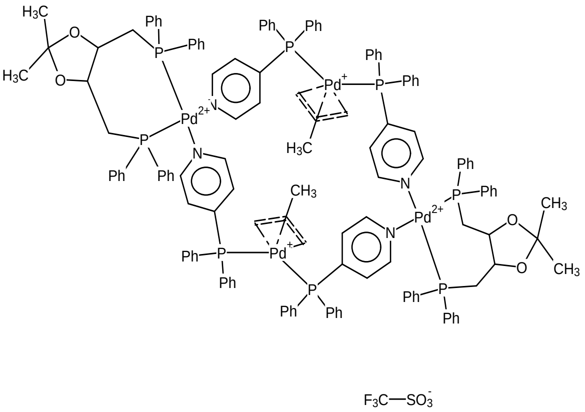

NIMXOA

Reference: M.Ferrer, A.Gallen, M.Martinez, M.Rocamora, R.Puttreddy, K.Rissanen (2022) *Dalton Trans.* ,**51,**5913

Formula: $C_{138}\ H_{134}\ N_4\ O_4\ P_8\ Pd_2\ Pt_2\ ^{6+},6(C_1\ F_3\ O_3\ S_1\ ^{1-})$

Compound Name: tetrakis(μ-4-(diphenylphosphino)pyridine)-bis([(2,2-dimethyl-1,3-dioxolane-4,5-diyl)bis(methylene)]bis(diphenylphosphine))-bis(2-methylallyl)-tetra-palladium hexakis(trifluoromethanesulfonate) unknown solvate

Space Group:	Fddd	***Cell:***	***a***	34.134(0)	***b***	39.026(0)	***c***	54.220(1)
Space Group No.:	70	***(Å,°)***	α	90.00	β	90.00	γ	90.00

R-Factor (%): 15.37 ***Temperature(K):*** 120 ***Density(g/cm^3):*** 1.345

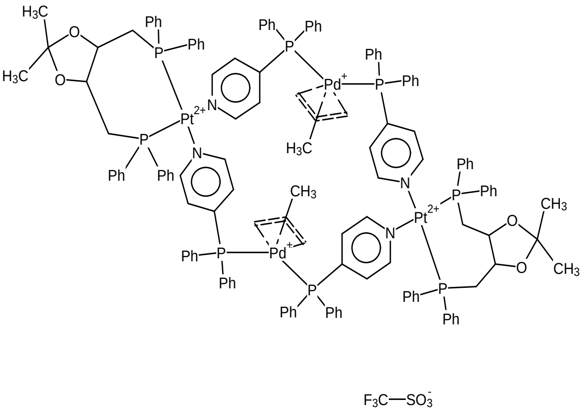

Search: search1 (Tue Nov 25 10:01:16 2025): Hits 13-16

NONYEU

Reference:	L.Hirsivaara, M.Haukka, J.Pursiainen (2001) *J.Organomet.Chem.* ,**633**,66
Formula:	$C_{42}\ H_{28}\ N_2\ O_8\ P_2\ W_2$
Compound Name:	bis(μ_2-Diphenyl(pyrid-4-yl)phosphine-N,P)-octacarbonyl-di-tungsten

Space Group:	P-1	***Cell:***	***a*** 8.645(0)	***b*** 9.186(0)	***c*** 12.733(0)		
Space Group No.:	2	***(Å,°)***	α 80.48(0)	β 89.21(0)	γ 82.94(0)		
R-Factor (%):	1.96	***Temperature(K):***	150	***Density(g/cm³):***	1.876		

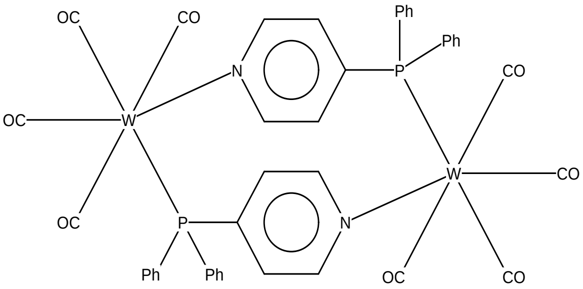

UDUGEG

Reference:	M.Kuil, P.E.Goudriaan, A.W.Kleij, D.M.Tooke, A.L.Spek, P.W.N.M.van Leeuwen, J.N.H.Reek (2007) *Dalton Trans.* ,2311
Formula:	$C_{101}\ H_{117}\ Cl_1\ N_6\ O_4\ P_2\ Pd_1\ Zn_2$
Compound Name:	(μ_2-1,2,4,5-tetrakis(N-(4,6-Di-t-butylsalicylidenealdiminato))benzene)-bis(μ_2-diphenyl(4-pyridyl)phosphine-N,P)-chloro-methyl-palladium-di-zinc(ii) unknown solvate

Space Group:	C2/c	***Cell:***	***a*** 32.664(1)	***b*** 12.443(1)	***c*** 25.374(1)
Space Group No.:	15	***(Å,°)***	α 90.00	β 97.31(0)	γ 90.00
R-Factor (%):	8.59	***Temperature(K):***	150	***Density(g/cm³):***	1.178

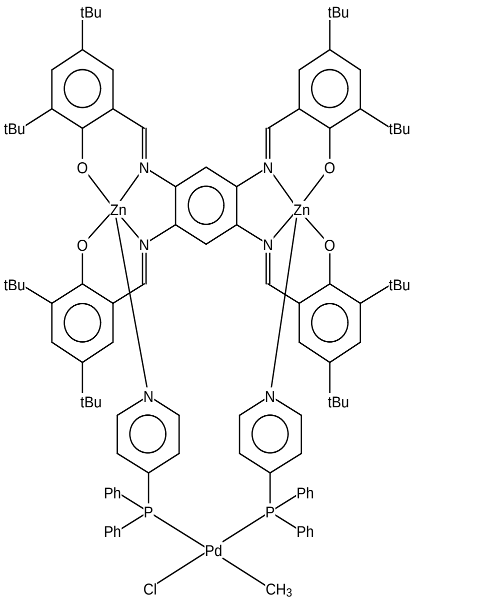

WERFIL

Reference:	M.Yamamoto, T.Nakanishi, Y.Kitagawa, T.Seki, Hajime Ito, Koji Fushimi, Y.Hasegawa (2018) *Bull.Chem.Soc.Jpn.* ,**91**,6
Formula:	$C_{49}\ H_{31}\ Eu_1\ F_{18}\ N_2\ O_8\ P_2$
Compound Name:	bis(diphenyl(4-pyridyl)phosphine oxide)-tris(1,1,1,5,5,5-hexafluoroacetylacetonato)-europium

Space Group:	P-1	***Cell:***	***a*** 13.061(0)	***b*** 13.696(0)	***c*** 15.495(1)
Space Group No.:	2	***(Å,°)***	α 76.17(0)	β 83.70(0)	γ 78.58(0)
R-Factor (%):	3.62	***Temperature(K):***	123	***Density(g/cm³):***	1.680

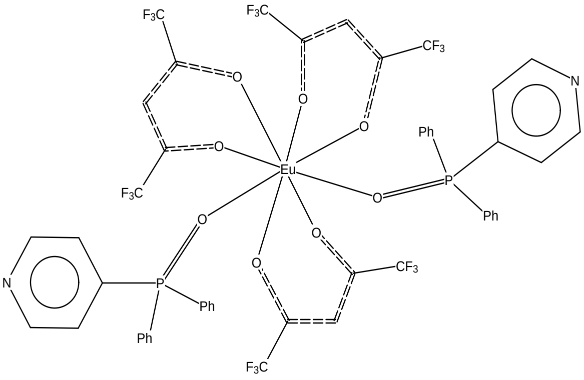

WERFOR

Reference:	M.Yamamoto, T.Nakanishi, Y.Kitagawa, T.Seki, Hajime Ito, Koji Fushimi, Y.Hasegawa (2018) *Bull.Chem.Soc.Jpn.* ,**91**,6
Formula:	$C_{34}\ H_{28}\ Cl_2\ N_2\ O_2\ P_2\ Pd_1,2(C_1\ H_4\ O_1)$
Compound Name:	dichloro-bis(4-(diphenylphosphoryl)pyridine)-palladium(ii) methanol solvate

Space Group:	P21/c	***Cell:***	***a*** 18.466(1)	***b*** 7.061(0)	***c*** 14.441(1)
Space Group No.:	14	***(Å,°)***	α 90.00	β 104.34(0)	γ 90.00
R-Factor (%):	5.76	***Temperature(K):***	123	***Density(g/cm³):***	1.456

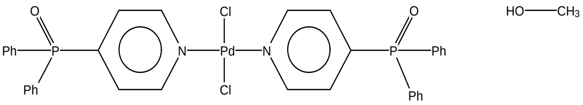

Search: search1 (Tue Nov 25 10:01:16 2025): Hits 17-18

XUWHOP

Reference: M.Ferrer, A.Gallen, A.Gutierrez, M.Martinez, E.Ruiz, Y.Lorenz, M.Engeser (2020) *Chem.-Eur.J.* ,**26,**7847

Formula: $C_{390} H_{336} N_{12} P_{24} Pd_6 Pt_6$ $^{18+}$,12($F_2 O_2 P_1$ $^{1-}$),6($F_6 P_1$ $^{1-}$)

Compound Name: dodecakis(μ-4-(diphenylphosphino)pyridine)-tris(μ-benzene-1,2,4,5-tetrayltetrakis(diphenylphosphine))-hexakis(2-methylallyl)-hexa-palladium-hexa-platinum hexakis(hexafluorophosphate) dodecakis(difluorophosphate) unknown solvate

Space Group:	C2/c	***Cell:***	***a*** 96.665(1)	***b*** 31.593(0)	***c*** 36.577(0)		
Space Group No.:	15	***(Å,°)***	α 90.00	β 94.53(0)	γ 90.00		
R-Factor (%):	13.86	***Temperature(K):***	100	***Density(g/cm³):***	1.172		

ZOZTER

Reference: V.Bocokic, M.Lutz, A.K.Burat, J.P.Lewtak, D.T.Gryko, A.Spek, J.N.H.Reek (2024) *CSD Communication(Private Communication) ,*

Formula: $C_{129} H_{126} N_9 O_8 P_1 Zn_1$

Compound Name: (2,3,9,10,16,17,23,24-octakis(4-t-butylphenoxy)-29H,31H-phthalocyaninato)-(4-(diphenylphosphanyl)pyridine)-zinc(ii) unknown solvate

Space Group:	P21/n	***Cell:***	***a*** 22.212(0)	***b*** 23.117(0)	***c*** 22.668(0)		
Space Group No.:	14	***(Å,°)***	α 90.00	β 90.92(0)	γ 90.00		
R-Factor (%):	8.78	***Temperature(K):***	125	***Density(g/cm³):***	1.157		

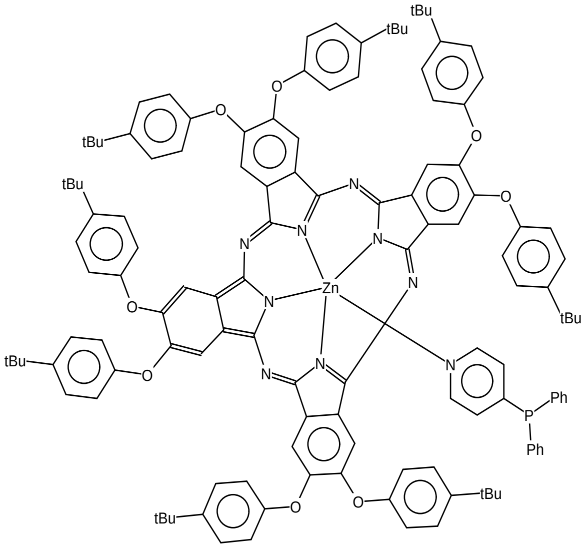

Supplement: Supplementary file 3 [file e-82-00096-sup3.pdf]
